# Health Tract, No. 260

**Published:** 1866-11

**Authors:** 


### IMMORTALITY'S DAWN.

One of the most impressive delineators ever listened to, was the Rev. David Nelson, M. D., while sitting under his ministry in our school-boy days. He had occasion to attempt a solution of the cause of the ineffable radiance that sometimes lights up the face of the dying christian after the power of speech has been lost. The illustration was afterwards repeated in his remarkable book on the "Cause and Cure of Infidelity."

The passage of the christian from the mortal to the immortal state, is like descending into a ditch, crossing the bottom, and climbing up on the other side; and just as he rises to the top the veil of eternity is gradually drawn aside, when he has his first view of the immortal state. It is then that the effulgent glories of the heavenly world so entrance his vision that he is wakened up to the new life before the old one is laid aside; and the sight so resplendent, impresses on the physical features some of the glories seen on Moses's face after he had been with Divinity; repeated again on the Mount of Transfiguration, when after communion with the Holy One, the Master's face "did shine as the snn." Even "his raiment became shining, exceeding white as snow, so as no fuller on earth can white them." In speaking of this "great mystery" a nameless writer says, "No one who passes the charmed boundery comes back to tell. The imagination visits the realms of shadows, sent out from some window in the soul, but wings its way back, with only an olive leaf in its beak, as a token of emerging life beyond the closelv bending horizon. The great sun comes and goes in the heavens, yet breathes no secret of the etherial wilderness; the crescent moon cleaves her nightly passage across the upper deep, but tosses over-board no signals. The sentinel stars challenge each other as they make their nightly rounds, but we catch no syllable of the countersign which gives passage into the heavenly camp. Between this and the other life is a great gulf fixed, across which neither eye nor foot can trace. The gentle friend whose eye we closed in the last sleep long years ago, died with rapture in her wonder-stricken eyes, a smile of ineffable joy on her lips, and hands folded over a triumphant heart; but she spoke no word, and intimated nothing of the vision which enthralled her."

Now and then, once in a generation it may be, one is permitted to return to life after having a glimpse of the other side. But their lips seem sealed, as if the subject was too holy for human converse. Whatever of sight was seen or sound was heard it was only described as a "glory unutterable."

